# Transcriptomic analysis reveals the function of m6A regulators in aged cochlea

**DOI:** 10.1016/j.bjorl.2025.101578

**Published:** 2025-04-07

**Authors:** Yanbing Lai, Bo Su, Xiaodi Wang, Chenghui Zeng, Hanqi Chu, Liangqiang Zhou, Dan Bing

**Affiliations:** Tongji Hospital Affiliated to Tongji Medical College of Huazhong University of Science and Technology, Department of Otorhinolaryngology Head and Neck Surgery, Wuhan, China

**Keywords:** Aged cochlea, m6A regulators, Transcriptomics, Mice, Immune-related genes

## Abstract

**Objective:**

Presbycusis is a prevalent health issue among the elderly. Previous studies have shown mechanisms related to this condition, but the underlying mechanisms of presbycusis remain elusive. N6-methyladenosine (m6A) modification in regulating gene expression and cellular functions has been implicated in the development of various diseases. Nevertheless, the potential role of m6A regulators in presbycusis is still unclear. In this study, we aim to determine the expression of m6A regulators in the cochleae of young and old mice, and to investigate their potential role in aging.

**Methods:**

We sequenced the transcriptome from the cochleae of six young (2-mo) and six old mice (24-mo) bioinformatics analysis. Differential expression analysis and downstream functional analysis was performed to identify m6A regulators. Association of m6A regulators with protein-protein interaction and transcription factor-miRNA networks were constructed to explore their regulatory mechanisms.

**Results:**

ALKBH5 and YTHDC1 were found upregulated in the aged cochleae. They were strongly correlated with immune-related pathways, immune molecular subtypes, and immune infiltration levels in old mice, suggesting their potential involvement in immune-related mechanisms of presbycusis. Receiver Operating Characteristic (ROC) curve analysis demonstrated the high diagnostic potential molecules of AlkB Homolog 5 (ALKBH5) and YTHDC1.

**Conclusion:**

This study has established a molecular foundation and introduce a novel perspective on the role of m6A regulators in presbycusis, emphasizing ALKBH5 and YTHDC1 as potential markers.

**Level of evidence:**

Acknowledging methodological similarities with Level 3 (non-randomized controlled cohort or case-control studies) in clinical research, we reference Level 3 as a comparative framework, while recognizing the distinct differences between clinical and animal research settings.

## Highlights

• ALKBH5 and YTHDC1 are potential useful diagnostic markers for cochlear aging.
• ALKBH5 and YTHDC1 are highly associated with immune pathways.
• ALKBH5 and YTHDC1 are highly associated with immune cell infiltrate.

## Introduction

Presbycusis is the most prevalent adult auditory deficiency, with a high incidence in the aged population.^1^, ^2^ Its rate in the elderly have steadily risen in recent years.^3^ Severe hearing loss contributes to social isolation, depression, frailty, dementia, and imposes an enormous economic burden on society.^4^, ^5^, ^6^, ^7^, ^8^ Presbycusis originates from the progressive aging of the cochlea. Thus, a better understanding of the underlying molecule and biochemical mechanisms in aged cochlea is essential for improving the life quality of elderly. Previous studies have shown that oxidative stress, inflammation, and genetic factors are closely related to aged cochlea.^1^, ^9^, ^10^, ^11^, ^12^, ^13^, ^14^, ^15^ However, few studies have investigated the function of m6A regulators in aged cochleae.

m6A methylation is prevalent and important in Ribonucleic Acid (RNA) modification and is involved in various cellular processes such as immune response, apoptosis, circadian rhythm, and the development of diverse diseases, such as obesity and cancer.^16^, ^17^, ^18^, ^19^, ^20^, ^21^, ^22^, ^23^, ^24^ m6A RNA methylation is conducted by a core protein complex, which comprises Methyltransferase-Like-3 (METTL3), Methyltransferase-Like-14 (METTL14) and Wilms’ Tumor 1-Associated Protein (WTAP).^25^, ^26^, ^27^, ^28^ Demethylation is mediated by two demethylases, including Fat-mass and Obesity-associated protein (FTO) and AlkB Homolog-5 (ALKBH5).^29^, ^30^ The biological effect of m6A modification is determined by reader proteins, which mainly includes YT521-B Homology (YTH) family proteins and insulin-like growth factor-2 mRNA-binding proteins.^31^, ^32^, ^33^, ^34^, ^35^ One study has shown that upregulated or downregulated METTL3 could alleviate or accelerate senescent phenotypes in human mesenchymal stem cells.^36^ However, the role of m6A regulators in aged cochleae is still unclear.

To address this question, we analyzed the expression of m6A regulators in the cochleae of C57BL/6J mice at two age points: 2-mo (young) and 24-mo (old). Through bioinformatics analysis, we predicted the potential functions of these regulators. RT-PCR experiments, using transcripts from these mice, verified our findings, revealing upregulation of ALKBH5 and YTHDC1 in the older mice. These regulators exhibited the capacity to differentiate between the two age groups. Additionally, they correlated with the expression of immune-related genes. The results indicated that m6A regulators are associated with cochlear aging and suggested a role of ALKBH5 and YTHDC1 in immune infiltration. These findings offer promising directions for future research on presbycusis.

## Methods

### Ethics approval and informed consent

The animal study was reviewed and approved by the Huazhong University of Science and Technology Ethics Committee.

### Data acquisition

We analysed expression profiles in the cochleae of six old (24-mo) and six young (2-mo) C57BL/6J mice from Beijing Vital River Laboratory using transcriptome sequencing. Mice were anesthetized with CO_2_, decapitated, and cochleae swiftly isolated under a microscope. Excess tissues were meticulously removed, and samples were frozen in liquid nitrogen. OE Biotech Co. Ltd. (Shanghai) conducted sequencing on an Illumina NovaSeq 6000 platform (PE150), streamlining the process.

### Animal ABR detection

Mouse hearing was evaluated using Auditory Brainstem Response (ABR) via tone burst tests. Mice were anesthetized with 8% chloral hydrate (5 μL/g IP) and maintained at normal temperature on a heat plate. In a soundproof chamber, mice underwent testing with pre-calibrated speakers emitting 5 ms pure tone pulses (1.5 ms cosine-squared envelope) at 21 Hz. Responses were amplified 20×, bandpass filtered (300‒3000 Hz), and averaged 500 times per intensity (90‒10 dB). The custom system (GAT-ABR03; Shenzhen Giant) recorded waveforms, determining the lowest repeatable response threshold for each frequency. Hearing thresholds were compared between young (n = 6) and old (n = 6) mice groups to assess hearing differences.

### Differential and correlational analysis of m6A regulators

A total of 24 m6A regulators were extracted from transcriptome sequencing data: including 8 writers (METTL3, METTL14, METTL16, WTAP, ZC3H13, RBM15, RBM15B, CBLL1), and 14 readers (YTHDC1, YTHDC2, YTHDF1, YTHDF2, YTHDF3, HNRNPC, FMR1, LRPPRC, HNRNPA2B1, IGF2BP1, IGF2BP2, IGF2BP3, RBMX, ELAVL1) and 2 erasers (FTO, ALKBH5). Wilcoxon test was used to find differentially expressed m6A regulators. Pearson’s correlation analysis was performed to determine correlations between different m6A regulators.

### Differential gene expression analysis and identification of immune-related genes

Differentially expressed genes (DEGs) were determined using the limma *R* package according to the screening criteria (adj.P-value < 0.05, |log2FC| > 1).^37^ Results of differential gene expression analysis were plotted using ggplot2 package and heatmap package.^38^, ^39^ The list of immune genes was obtained from the Import database in the InnateDB website (https://www.innatedb.com/), which contains 4815 immune-related genes.^40^ Immune-related DEGs were extracted according to the list of immune genes and the shared genes from DEGs.

### GO, KEGG, GSEA and GSVA

Gene Ontology (GO) enrichment analysis identified enriched biological functions in genes of interest, while Kyoto Encyclopedia of Genes and Genomes (KEGG) pathway analysis revealed enriched pathways. Both analyses employed clusterProfiler with adj.P < 0.05.^41^, ^42^, ^43^ Gene Set Enrichment Analysis (GSEA), a popular method for genome-wide microarray data, compared genes to predefined sets using clusterProfiler (*p* < 0.05). Gene Set Variation Analysis (GSVA) assessed enrichment of specific gene sets, utilizing the GSVA package with KEGG v7.4 as reference and Gaussian distribution. Differentially expressed pathways were selected via limma.

### Protein-protein interaction network and transcription factor-miRNA network

For differentially expressed m6A regulators, Search Tool for the Retrieval of Interacting Genes (STRING, http://www.string-db.org/, Version: 11.0) and Cytoscape software were used to build an interaction network.^44^, ^45^ To further analyse the networks of transcription factors and small molecule compounds, we uploaded four differentially expressed m6A regulators to NetworkAnalyst (https://www.networkanalyst.ca/) and used TF-gene interactions and Gene-miRNA interactions modules for analysis.^46^ The reference databases were the TF database from ENCODE (http://cistrome.org/BETA/) and miRTarBase (https://mirtarbase.cuhk.edu.cn, Version: 8.0). The Cytoscape software was used to visualize the resulting interaction network.

### Construction of immune-related gene clusters

Consensus clustering, facilitated by the ConsensusClusterPlus *R* package, identified immune-related DEGs and determined the optimal number of clusters (K) through 1000 iterations and 80% sample resampling.^47^ The resampling method validated clustering reasonability. The optimal *K* was chosen based on the Cumulative Distribution Function (CDF) curve clustering scores and the Area Under the Curve (AUC) shape. Pearson’s test assessed the correlation between m6A regulators and molecular subtypes.

### Analysis of immune cell infiltration

The online tool CIBERSORTx (https://cibersortx.stanford.edu/) was used to calculate sample immune cell infiltration situation.^48^ The differences of the degree of immune infiltration were calculated by Wilcoxon test. The correlation among the infiltration degree of different immune cells was analysed. Finally, the correlation among immune cells and differentially expressed m6A regulators were also analysed.

### Validation of m6A regulators with ability to discriminate mice age

We adopted a logistic regression algorithm to analyse the relevance of differentially expressed m6A regulators and age-related grouping. The fitting result was reported using the Receiver Operating Characteristic (ROC) curve.

### Validation of cochlear tissue expression by quantitative polymerase chain reaction (qPCR)

The study involved 15 mice per age group (young and old), with each sample comprising 6 mouse cochleae. Total RNA was extracted from mouse cochleae using TRIzol (Takara), followed by cDNA synthesis from 1 μg RNA using HiScript® II Q RT SuperMix (Vazyme). qPCR was performed with ChamQ Universal SYBR qPCR Master Mix (Vazyme), normalizing to β-actin. Ct values were averaged and analyzed using the 2^-ΔΔCt^ method. Primer sequences are listed in Table 1.

**Table 1:** Primer sequence.

Gene	Forward primer (5’ to 3’)	Reverse primer (5’ to 3’)
*ALKBH5*	CGCGGTCATCAACGACTACC	CGCGGTCATCAACGACTACC
*CBLL1*	TCAGCCCGTGGTATCTCAC	GGTGGTGCGTAATGTTGCT
*ELAVL1*	GGATGACATTGGGAGAACGAAT	TGTCCTGCTACTTTATCCCGAA
*FMR1*	CAATGGCGCTTTCTACAAGGC	TCTGGTTGCCAGTTGTTTTCA
*FTO*	TTCATGCTGGATGACCTCAATG	GCCAACTGACAGCGTTCTAAG
*HNRNPA2B1*	CAGGGTAGTTGAGCCAAAACG	TTCCAGACTGCCTATCGGTAA
*HNRNPC*	CATTGGGAATCTCAACACTCTGG	CAAATGAGGAACCGTACATCTCC
*LRPPRC*	GTAGCCCAGGGAGCAATCAAG	GACACCTAACTGCTGAAGTTTGT
*METTL14*	CTGAGAGTGCGGATAGCATTG	GAGCAGATGTATCATAGGAAGCC
*METTL16*	GACAAACCACCTGACTTCGCA	TCTGACTGCTTCGGGGTCTT
*METTL3*	CTGGGCACTTGGATTTAAGGAA	TGAGAGGTGGTGTAGCAACTT
*RBM15*	CGAGTCCGCTGTGTGAAAC	TCCCCACGAGAACTGGAGTC
*RBM15B*	AGGGCGAAGGTGGCTATGT	GCGAGGTGTTAGGTCCGAG
*RBMX*	AGAGACGAATGAGAAAGCCCT	AGTGACAAAAGCGAATCCTCTTG
*WTAP*	GAACCTCTTCCTAAAAAGGTCCG	TTAACTCATCCCGTGCCATAAC
*YTHDC1*	GTCCACATTGCCTGTAAATGAGA	GGAAGCACCCAGTGTATAGGA
*YTHDC2*	GAAGATCGCCGTCAACATCG	GCTCTTTCCGTACTGGTCAAA
*YTHDF1*	ACAGTTACCCCTCGATGAGTG	GGTAGTGAGATACGGGATGGGA
*YTHDF2*	GAGCAGAGACCAAAAGGTCAAG	CTGTGGGCTCAAGTAAGGTTC
*YTHDF3*	CATAGGGCAACAGAGGAAACAG	ATCTCCAGCCGTGGACCAT
*ZC3H13*	ATCCCGAAGACCTAGCGTATT	TGAAGGGCCATGTATGAACCT
*IGF2BP1*	CGGCAACCTCAACGAGAGT	GTAGCCGGATTTGACCAAGAA
*IGF2BP2*	GTCCTACTCAAGTCCGGCTAC	CATATTCAGCCAACAGCCCAT
*IGF2BP3*	CCTGGTGAAGACGGGCTAC	TCAACTTCCATCGGTTTCCCA

### Statistical analysis

Statistical analyses were performed in *R* programming (https://www.r-project.org, Version: 4.1.0), applying the Benjamini-Hochberg method for multiple testing corrections and FDR to minimize false positives. Independent *t*-tests and Mann-Whitney *U* tests assessed normally and non-normally distributed variables, respectively. Logistic regression models evaluated diagnostic potential of m6A regulators, with ROC curves plotted using pROC R package. All tests were two-sided, and *p* < 0.05 indicated statistical significance.

## Results

### Audiological manifestations in young and old mice

**Fig. 1:**
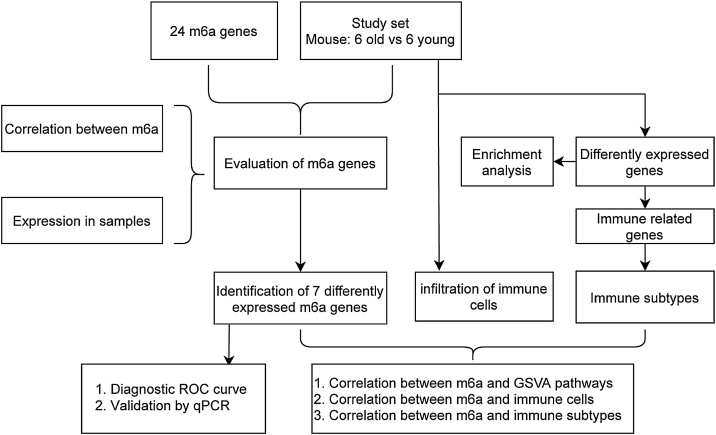
The flow chart of this study.

**Fig. 2:**
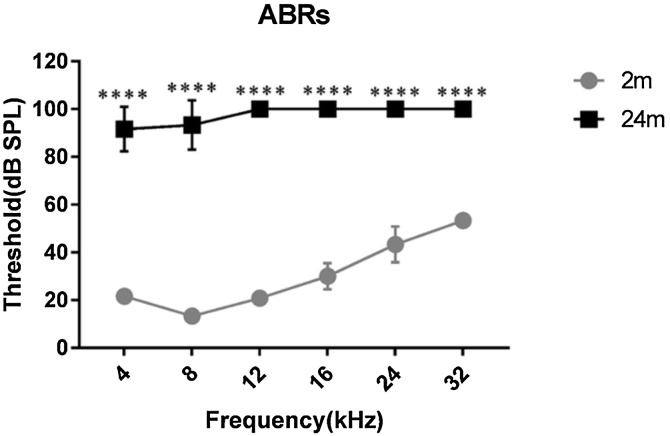
The mice hearing threshold on 4‒32 kHz frequency in toneburst-evoked auditory brainstem response.

Fig. 1 outlines the study design. Auditory brainstem responses of 2- and 24-mo mice confirmed auditory function. Assessment of ABR thresholds revealed that 2-mo-old mice possess normal hearing, whereas 24-mo-old mice exhibited severe-to-profound hearing impairment (Fig. 2), to the extent that the ABR was barely detectable even at the maximum sound output setting of the equipment (90 dB SPL). This highlighted a significant discrepancy in auditory function between the two age groups.

### Differential expression and correlation analysis of m6A methylation regulators

**Fig. 3:**
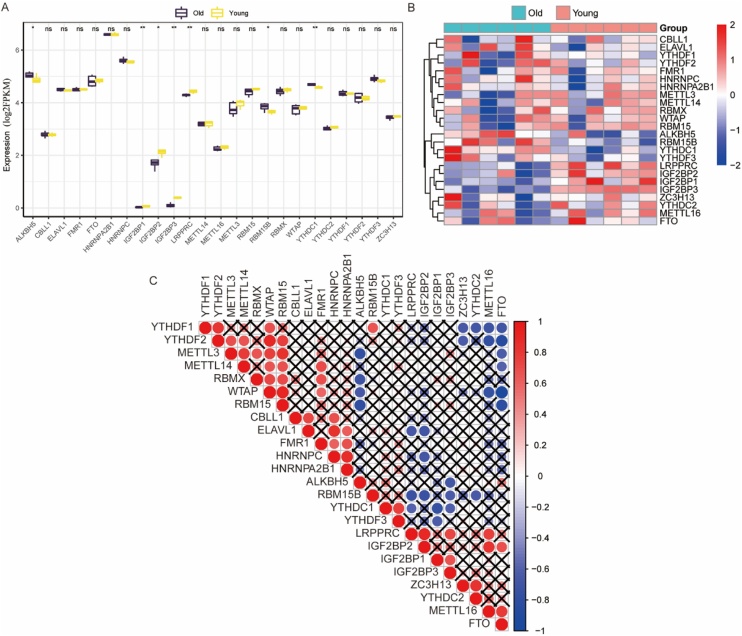
Differential and correlational analysis of m6A regulators. (A) The mRNA expression levels of m6A regulators in the old and young groups. The difference between the groups was tested by Wilcoxon. *p < 0.05, **p < 0.01; ns: not significant. (B) The heatmap of mRNA expression levels of m6A regulators across different samples. (C) Correlations among the expression of m6A regulators. X: not significant. (m6A, N6-methyladenosine).

**Fig. 4:**
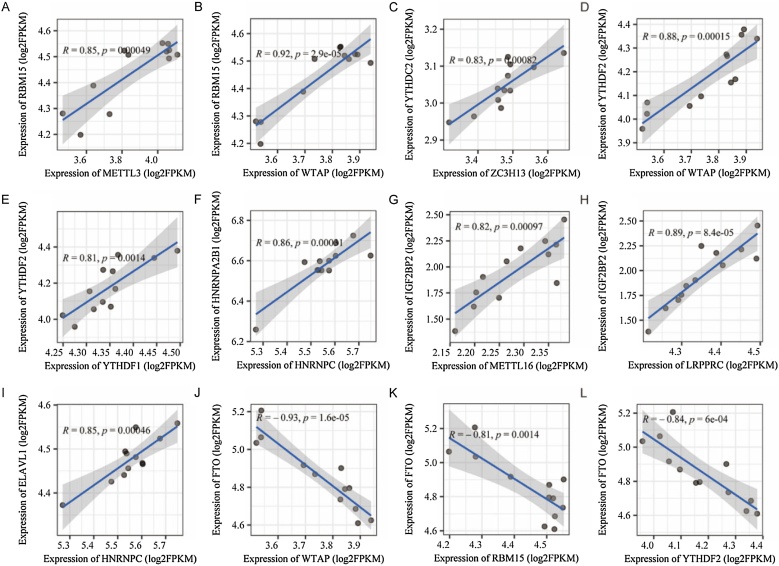
Dot/line plot of Correlation analysis among the expression of m6A regulators. (A‒L) These correlations among the expression of m6A regulators were significant. (m6A, N6-methyladenosine).

Seven differentially expressed m6A regulators were identified in old mice, with ALKBH5, RBM15B, and YTHDC1 upregulated, and LRPPRC, IGF2BP1, IGF2BP2, IGF2BP3 downregulated compared to young mice (Fig. 3A‒B). Correlation analysis of 24 m6A regulators revealed strong positive and negative associations (Figs. 3C and 4 ). Age-stratified analysis maintained most correlations, excluding age-related bias (Supplementary Fig. 1).

### Identification of differentially expressed genes

**Fig. 5:**
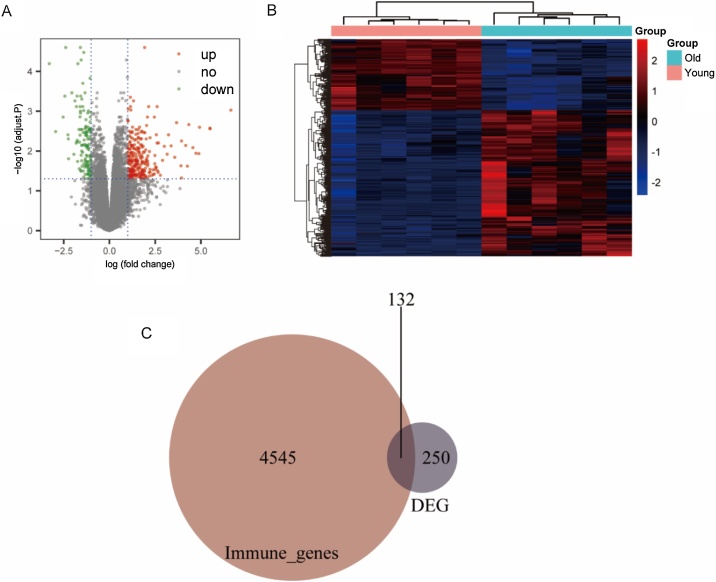
Identification of differentially expressed genes and immune-related genes. (A‒B) Volcano and heat maps of differential expression analysis. (C) Venn diagram of common genes in DEGs and immune-related genes. (DEGs, Differentially Expressed Genes).

We found 257 genes with upregulated expression and 125 genes with downregulated expression in old vs young. These DEGs are presented in a volcano plot and heatmap (Fig. 5A‒B). We also identified 132 immune-related gene from these DEGs using Venn diagram analysis and these immune-related genes were used for further analysis (Fig. 5C).

### Functional enrichment analysis

**Fig. 6:**
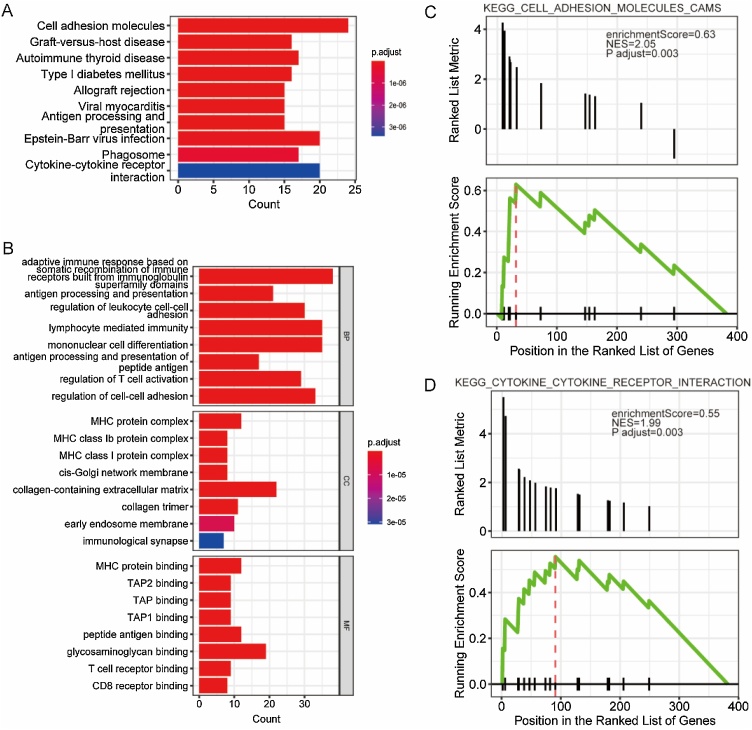
KEGG, GO and GSEA analysis of DEGs. (A) Bar chart of KEGG analysis. (B) Bar chat of GO analysis in BP, CC and MF categories. (C‒D) DEGs mainly enriched in two pathways by GSEA analysis. (KEGG, Kyoto Encyclopedia of Genes and Genomes; GO, Gene Ontology; GSEA, Gene Set Enrichment Analysis; DEGs, differentially expressed genes; BP, Biological Processes; CC, Cellular Component; MF, Molecular Function).

**Fig. 7:**
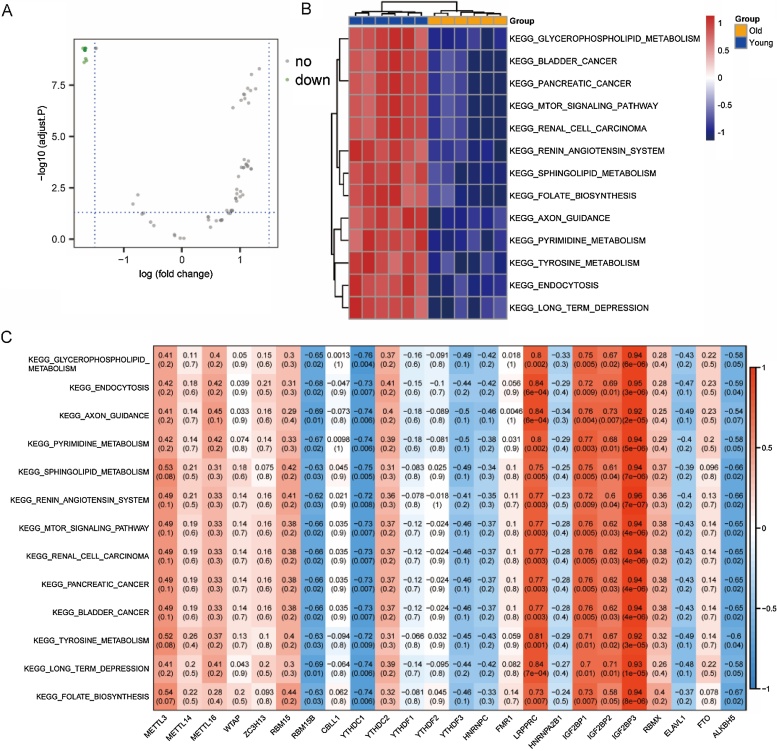
GSVA of DEGs. (A) Volcano plot of differential pathways by GSVA. (B) Heatmap of enrichment score of differential pathways in different samples. (C) The correlation among differential pathways and the expression level of m6A regulators. (GSVA, Gene Set Variation Analysis).

KEGG analysis revealed DEG enrichment in immune-related pathways, including cell adhesion, autoimmune diseases, and allograft rejection (Fig. 6A, Table 2). GO enrichment analysis showed that DEGs were involved in adaptive immune response, antigen processing, and leukocyte adhesion (BP); MHC protein Complexes (CC); and MHC protein binding (MF) (Fig. 6B, Table 3). GSEA identified enriched pathways related to cell adhesion and cytokine receptor interaction (Fig. 6C‒D). GSVA results indicated DEG aggregation in glycerophospholipid metabolism, endocytosis, axon guidance, pyrimidine metabolism, and sphingolipid metabolism (Fig. 7A‒B). Correlation analysis between m6A regulators and GSVA scores showed that LRPPRC, IGF2BP1, IGF2BP2 and IGF2BP3 were positively associated, while RBM15B, YTHDC1, and ALKBH5 were negatively associated with these pathways (Fig. 7C).

**Table 2:** The result of KEGG pathway analysis.

ID	Description	GeneRatio	p.adjust	Count
mmu04514	Cell adhesion molecules	24/158	5.18E-13	24
mmu05332	Graft-versus-host disease	16/158	6.86E-13	16
mmu05320	Autoimmune thyroid disease	17/158	1.27E-12	17
mmu04940	Type I diabetes mellitus	16/158	2.09E-12	16
mmu05330	Allograft rejection	15/158	5.62E-12	15
mmu05416	Viral myocarditis	15/158	8.32E-10	15
mmu04612	Antigen processing and presentation	15/158	9.99E-10	15
mmu05169	Epstein-Barr virus infection	20/158	8.23E-08	20
mmu04145	Phagosome	17/158	3.82E-07	17
mmu04060	Cytokine-cytokine receptor interaction	20/158	3.41E-06	20

**Table 3:** The result of gene ontology analysis.

Ontology	ID	Description	GeneRatio	p.adjust	Count
BP	GO:0002460	Adaptive immune response based on somatic recombination of immune receptors built from immunoglobulin superfamily domains	38/335	3.40E-17	38
BP	GO:0019882	Antigen processing and presentation	21/335	2.58E-15	21
BP	GO:1903037	Regulation of leukocyte cell-cell adhesion	30/335	3.45E-15	30
BP	GO:0002449	Lymphocyte mediated immunity	35/335	3.45E-15	35
BP	GO:1903131	Mononuclear cell differentiation	35/335	6.01E-15	35
BP	GO:0048002	Antigen processing and presentation of peptide antigen	17/335	1.40E-14	17
BP	GO:0050863	Regulation of T cell activation	29/335	2.54E-14	29
BP	GO:0022407	Regulation of cell-cell adhesion	33/335	2.54E-14	33
BP	GO:0022409	Positive regulation of cell-cell adhesion	27/335	2.54E-14	27
BP	GO:0002683	Negative regulation of immune system process	33/335	2.54E-14	33
CC	GO:0042611	MHC protein complex	12/333	2.50E-12	12
CC	GO:0032398	MHC class Ib protein complex	8/333	2.40E-08	8
CC	GO:0042612	MHC class I protein complex	8/333	3.33E-08	8
CC	GO:0033106	cis-Golgi network membrane	8/333	6.66E-08	8
CC	GO:0062023	Collagen-containing extracellular matrix	22/333	7.50E-08	22
CC	GO:0005581	Collagen trimer	11/333	1.07E-07	11
CC	GO:0031901	Early endosome membrane	10/333	7.89E-06	10
CC	GO:0001772	Immunological synapse	7/333	3.09E-05	7
CC	GO:0005769	Early endosome	16/333	3.47E-05	16
CC	GO:0032421	Stereocilium bundle	8/333	3.47E-05	8
MF	GO:0042287	MHC protein binding	12/327	1.48E-09	12
MF	GO:0046979	TAP2 binding	9/327	1.48E-09	9
MF	GO:0046977	TAP binding	9/327	1.48E-09	9
MF	GO:0046978	TAP1 binding	9/327	1.48E-09	9
MF	GO:0042605	Peptide antigen binding	12/327	1.48E-09	12
MF	GO:0005539	Glycosaminoglycan binding	19/327	2.07E-09	19
MF	GO:0042608	T cell receptor binding	9/327	6.78E-09	9
MF	GO:0042610	CD8 receptor binding	8/327	1.59E-08	8
MF	GO:0030881	Beta-2-microglobulin binding	8/327	2.03E-08	8
MF	GO:0042277	Peptide binding	22/327	2.15E-08	22

### PPI network, transcription factors and miRNA analysis

**Fig. 8:**
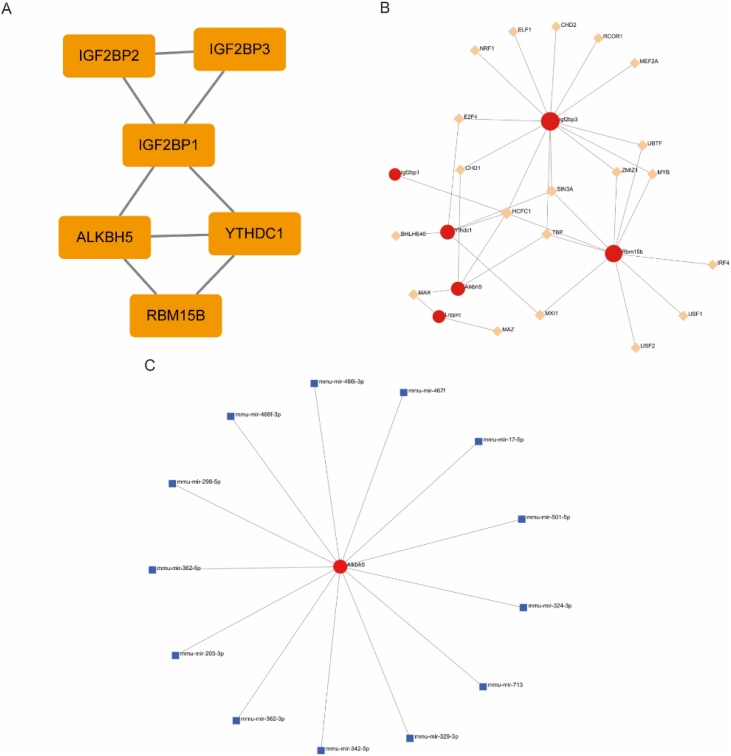
Interaction analysis of differentially expressed m6A regulators. (A) The protein-protein interactions among differentially expressed m6A regulators. (B) Interaction network between differentially expressed m6A regulators and transcription factors. (C) Interaction network between differentially expressed m6A regulators and miRNA. (m6A, N6-methyladenosine).

To delve into the interplay among six differentially expressed m6A regulators, we conducted a comprehensive Protein-Protein Interaction (PPI) network and transcription factor analysis (Fig. 8A‒B). Notably, RBM15B linked with 2, YTHDC1 with 5, ALKBH5 with 4, LRPPRC with 2, and IGF2BP3 with 13 transcription factors, showcasing their diverse regulatory roles. Furthermore, miRNA analysis revealed ALKBH5’s intricate interactions with 13 miRNAs (Fig. 8C), emphasizing its multifaceted regulatory landscape.

### Construction of immune-related clusters and correlation analysis

**Fig. 9:**
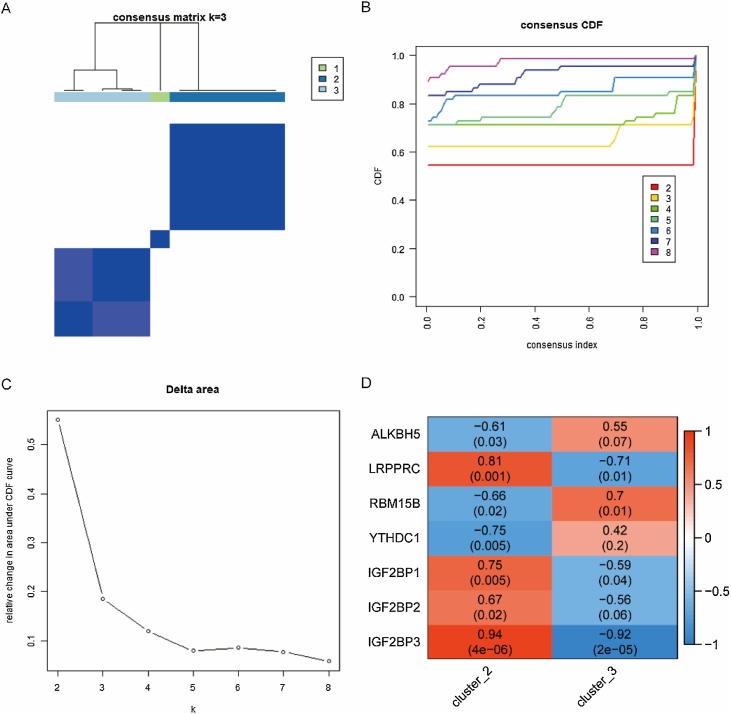
Immune-related clusters and correlation analysis. (A‒C) The process of constructing immune-related clusters. (D) The correlation among immune-related clusters and differentially expressed m6A regulators.

To investigate the link between differentially expressed m6A regulatory factors and immune-associated DEGs, we employed unsupervised consensus clustering on 132 immune-related DEGs, revealing three distinct immune molecular subtypes (Fig. 9A‒C). The optimal number of clusters was determined by analysing the curve area under the consensus distribution function. Notably, correlation analysis highlighted a strong association between the m6A regulators and immune subtypes 2 and 3 (Fig. 9D).

### Immune cell infiltrate analysis and correlation analysis

**Fig. 10:**
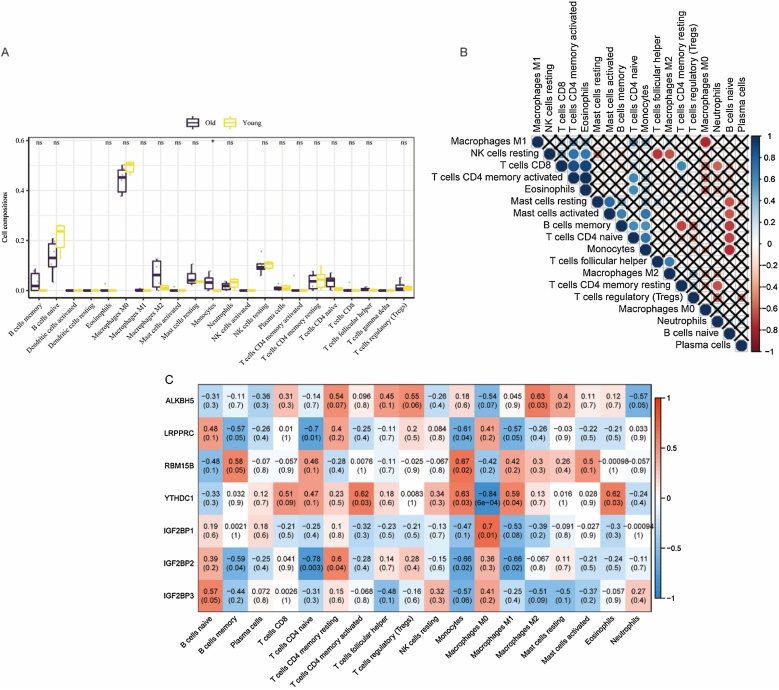
Analysis of immune cell infiltration. (A) The extent of immune cell infiltration in old and young group. The difference between the groups was tested by Wilcoxon. *p < 0.05; ns, not significant. (B) The correlation between different immune cells. X: not significant (C) The correlation among immune cells and differentially expressed m6A regulators.

Using CIBERSORT, we analyzed immune cell infiltration in young and old mouse groups (Fig. 10A), revealing heightened monocyte infiltration in the older group. Correlation analysis among immune cell infiltrations (Fig. 10B) and their associations with m6A regulators (Fig. 10C) showed intricate patterns. Notably, LRPPRC negatively correlated with naïve CD4+ T-cells and monocytes, while ALKBH5 positively correlated with M2 macrophages. RBM15B positively correlated with monocytes, and YTHDC1 displayed mixed correlations, positively with activated CD4+ memory T-cells, monocytes, M1 macrophages, and eosinophils, but negatively with M0 macrophages. IGF2BP1 positively correlated with M0 macrophages, while IGF2BP2 positively associated with resting CD4+ memory T-cells and negatively with naïve CD4+ T-cells, monocytes, and M1 macrophages.

### Discrimination ability of differentially expressed m6A regulators for mice age

**Fig. 11:**
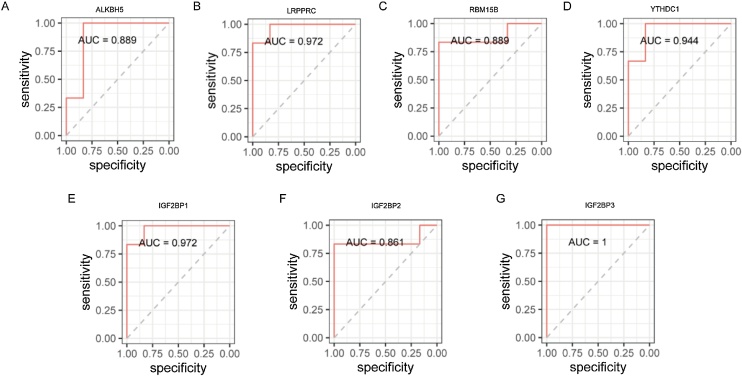
Discrimination ability of differentially expressed m6A regulators for mice age. (A‒G) ROC of 7 differentially expressed m6A regulators. (ROC, Receiver Operating Characteristic Curve).

To validate the relevance of differentially expressed m6A regulators to age-related grouping, we applied a logistic regression algorithm. These regulators demonstrated strong discriminatory power, achieving an ROC AUC over 0.85, effectively differentiating mouse ages (Fig. 11A‒G).

### Validation of m6A regulatory factor expression and localization of ALKBH5 in vivo

**Fig. 12:**
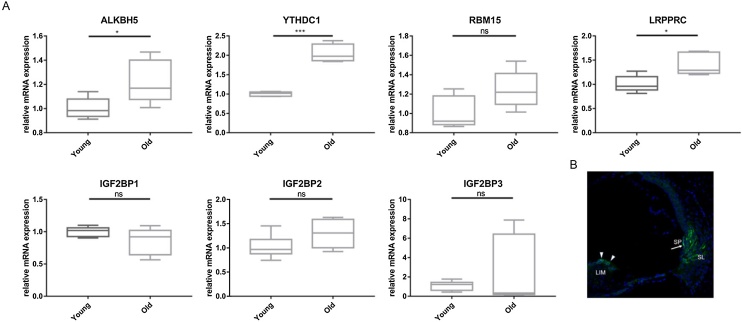
Validation of m6A regulatory factor expression and localization of ALKBH5 In Vivo. (A) The expression of m6A regulatory factors in cochlear tissue by qPCR. n = 5; *p < 0.05; **p < 0.01, ***p < 0.001; ns, not significant. (m6A, N6-methyladenosine; qPCR, differentially expressed genes). (B) Localization of ALKBH5 in mouse cochlea. (LIM, spiral limbus, white arrowhead points; SP, Spiral Prominence, white straight arrow points; SL, Spiral Ligament).

To validate m6A regulatory factor expression in the cochleae, we performed qPCR on total RNA extracted from young and old mouse cochleae (Fig. 12A). Our results corroborated the bioinformatics analysis for ALKBH5 and YTHDC1, showing similar trends. IGF2BP1 expression, though not significantly downregulated in old mice, mirrored the bioinformatics predictions. Additional qPCR data for other m6A regulators are provided in Supplementary Fig. 2. Furthermore, we validated the localization of ALKBH5 protein in the mouse cochleae, with fluorescent staining primarily observed in the spiral ligament, spiral prominence, and spiral limbus (Fig. 12B).

## Discussion

Presbycusis is characterized by bilateral symmetry, progressive sensorineural hearing loss that characterizes the aged cochlea. The C57BL/6 inbred mouse strain is commonly used as a model to simulate presbycusis. Numerous studies have confirmed that this strain of mice experiences sensorineural hearing loss during middle to old age, typically after reaching 6-mo of age. The hearing loss first manifests as a decline in high-frequency hearing, and subsequently, this impairment spreads to all frequency ranges. The 24-mo-old mice served in this study adequately represented advanced aging, exhibiting poorer hearing performance and thus serving as a more exemplary model.

Previous studies have shown mechanisms related to this condition, such as oxidative stress, inflammaging and genetic predispositions. The role of epigenetic modifications, particularly m6A methylation, remains unexplored. However, m6A methylation, regulates diverse biological process from gene expression to mRNA stability and translation, making it a potential key player in the pathogenesis of presbycusis.

Currently, only a few typical animal models that accurately recapitulate the complex, age-related process leading to presbycusis. But few animal studies have explored the therapeutic potential of modulating m6A methylation of basic research findings into clinical applications. Studying the role of m6A methylation in presbycusis provides new direction for our understanding of presbycusis.

Our approach involved confirming hearing loss in aged mice and identifying differentially expressed genes, particularly those encoding m6A regulators and immune-related proteins. Our study offers new insights into cochlear aging. For the first time, significant upregulation of ALKBH5 and YTHDC1 expression were observed in the cochleae of aged mice. In Parkinson’s disease mouse models, both gene expression and protein levels of ALKBH5 and IGF2BP2 in the substantia nigra were upregulated,^49^ which in consistent with the trend of ALKBH5 upregulation observed in aging cochleae, suggesting that ALKBH5 may play a significant role in geriatric disease. Since presbycusis shares a pathogenic basis with numerous geriatric diseases, including dementia, frailty, Alzheimer’s disease, and type 2 diabetes, a common feature of these diseases is inflammaging in their target organs.^50^ It can be inferred that alterations of ALKBH5 expression may be associated with a broad range of geriatric disease processes, particularly related to the mechanisms of inflammaging. Additionally, investigations into atherosclerosis have revealed that a reduction in YTHDC1 expression may exacerbate inflammatory responses, accelerate the expression of senescence-related genes, and thereby facilitate the development of atherosclerosis.^51^ This suggests that alterations in YTHDC1 expression may serve as potential targets for a variety of aging-related diseases. Moreover, another m6A regulator, METTL3, has been found to alleviate the senescent phenotype in human mesenchymal stem cells, which consistent with our findings in the mouse aging cochleae. These findings indicated that m6A modifications may indeed play a role in the aging process.

To explore how m6A regulatory factors regulate cochlear aging, our results were consistent with previous studies that documented the effects in immune function during aging, both in the innate and adaptive immune systems.^52^ We found that all differentially expressed m6A regulators are correlated with aging process, at least in part, through immune-related pathways.

Furthermore, we evaluated the immune microenvironment of old mouse cochleae and found that monocytes were the mostly enriched immune cell at relative levels. We also performed correlation analysis to investigate whether differentially expressed m6A regulatory factors were associated with immune cells. A series of studies have showed that m6A regulators were significantly associated with tumor immune microenvironment. For example, ALKBH5 inhibited the expansion and cytotoxicity of T-cell in intrahepatic cholangiocarcinoma.^49^ In addition, Shi et al. found that YTHDF1 facilitated tumor cell proliferation by regulating the translation efficiency of several immune checkpoints in non-small-cell lung cancer.^53^ Although cochlea was long considered as “immune privilege” due to blood-labyrinth barrier, which blocks the entry and establishment of immune cells in the cochlea. More recent work has clarified that a variety of immune cells are present in the mouse cochleae under normal physiological conditions.^54^ In our study, we found that YTHDC1 was positively associated with activated CD4^+^ memory T-cells, monocytes, M1 macrophages, and eosinophils and negatively correlated with M0 macrophages.^55^ IGF2BP2 was positively associated with resting CD4^+^ memory T-cells and negatively associated with naïve CD4^+^ T-cells, monocytes, and M1 macrophages. IGF2PB2 was reported to play an important role in macrophage activation.^56^ These data suggested that m6A regulatory factors can modulate the infiltration of immune cells, potentially triggering an immune response within the immune microenvironment, thereby influencing the clinical outcomes of presbycusis.

For the qPCR validation, we found that expression of ALKBH5 were consistent with the results of bioinformatics analysis. Additionally, we found that ALKBH5 protein mainly localized in the spiral ligament of the cochleae. This observation raises the question of whether the localized expression of ALKBH5 in the spiral ligament is associated with the inflammatory cell movement, which has been reported to vary with age in the study.^57^ Further research is needed to elucidate this potential correlation. Notably, the YTH domain family of proteins, including YTHDC1, YTHDC2, YTHDF1, YTHDF2, and YTHDF3, were upregulated in the cochleae of aged mice (Supplementary Fig. 2). m6A modification can be recognized by YTH domain family proteins in a methylation-dependent manner.^55^ In immunity, the YTH family plays an important role in antiviral immunity, inflammatory immunity, and anti-tumor immunity. Previous studies suggest that the YTH domain family is associated with the infiltration of macrophages.^55^ Alternatively, the loss of YTH domain family proteins in tumors can enhance the cytotoxic function of CD8^+^ T-lymphocytes, thereby accelerating tumor immune evasion.^53^ Also, YTH domain family is important for for glycolysis metabolism and changes in nutrition are also an important mechanism underlying metabolism immunity.^58^ Therefore, we suppose that YTH domain family proteins might be important in the cochlear aging process, further studies are needed to explore its function.

In the present study, there still exist some limitations. Firstly, the statistics we analysed were mainly from the databases, therefore, we should present more *in vitro* and *in vivo* experiments to identify the function and mechanisms of these m6A regulatory factors. Secondly, we only verified the m6A regulators at the mRNA level. Further studies are needed to determine the function of m6A regulatory factors at the protein level. Finally, the interaction between m6A regulators and immune pathways in the cochlear aging process needs to be further explored using cell and animal experiments.

## Conclusion

Our findings indicate that m6A regulatory factors likely play a pivotal role in the aging process of the cochlea. ALKBH5 and YTHDC1 emerge as promising biomarkers, presenting a potential pathway for diagnosing presbycusis. Furthermore, our study has established a molecular foundation by comparing young mice with normal hearing to old mice with severely impaired auditory functions, thereby enhancing our understanding of the mechanism underlying presbycusis. As a result, our research introduces a novel perspective and could potentially pave the way for strategies aimed at developing preventative therapies.

## CRediT authorship contribution statement

DB and LQZ carried out the study design. DB and YBL performed data acquisition and bioinformatic analysis. BS performed interpretation of data and wrote the manuscript. XDW performed qPCR and analysed the result. CHZ conducted extraction of mice cochleae. DB, LQZ and HQC revised the manuscript. All authors read and approved the final manuscript; agreed to submit to the current journal.

Contribution to the field statement: This study first investigated the role of m6A regulators in age-related hearing loss, which provided a new hypothesis to illustrate the process of cochlear aging. Furthermore, we found two promising biomarkers for this illness: ALKBH5 and YTHDC1. These two biomarkers are suitable for next functional and mechanism research, which provided specific molecular targets and research orientation for researchers in this filed.

## Funding

This study was supported by grants from the National Key R&D Program of China (2023YFC2508400), the Hubei Provincial Key Research and Development Program (2022BCA006), the Wuhan Knowledge Innovation Project (2022022101015011), the Key Project of Tongji Hospital Scientific Research Fund (2023A01), and the 10.13039/501100003819Hubei Provincial Natural Science Foundation (grant number 2022CFB282).

## Declaration of competing interest

The authors declare that the research was conducted in the absence of any commercial or financial relationships that could be construed as a potential conflict of interest.

## Data availability

The datasets used in current study are available from the corresponding author on reasonable request.
