# Supplementary material for: Transcriptomic analysis reveals the function of m6A regulators in aged cochlea
Source: Braz J Otorhinolaryngol. 2025 Apr 7;91(3):101578. doi: 10.1016/j.bjorl.2025.101578 (PMC12005922; doi:10.1016/j.bjorl.2025.101578)
Supplement: Supplementary file 1 [file mmc1.docx]

**BJORL-D-24-00357_ Supplementary Material**

**Supplementary Figure 1** Dot/line plot of Correlation analysis among the expression of m6A regulators in old and yang group.


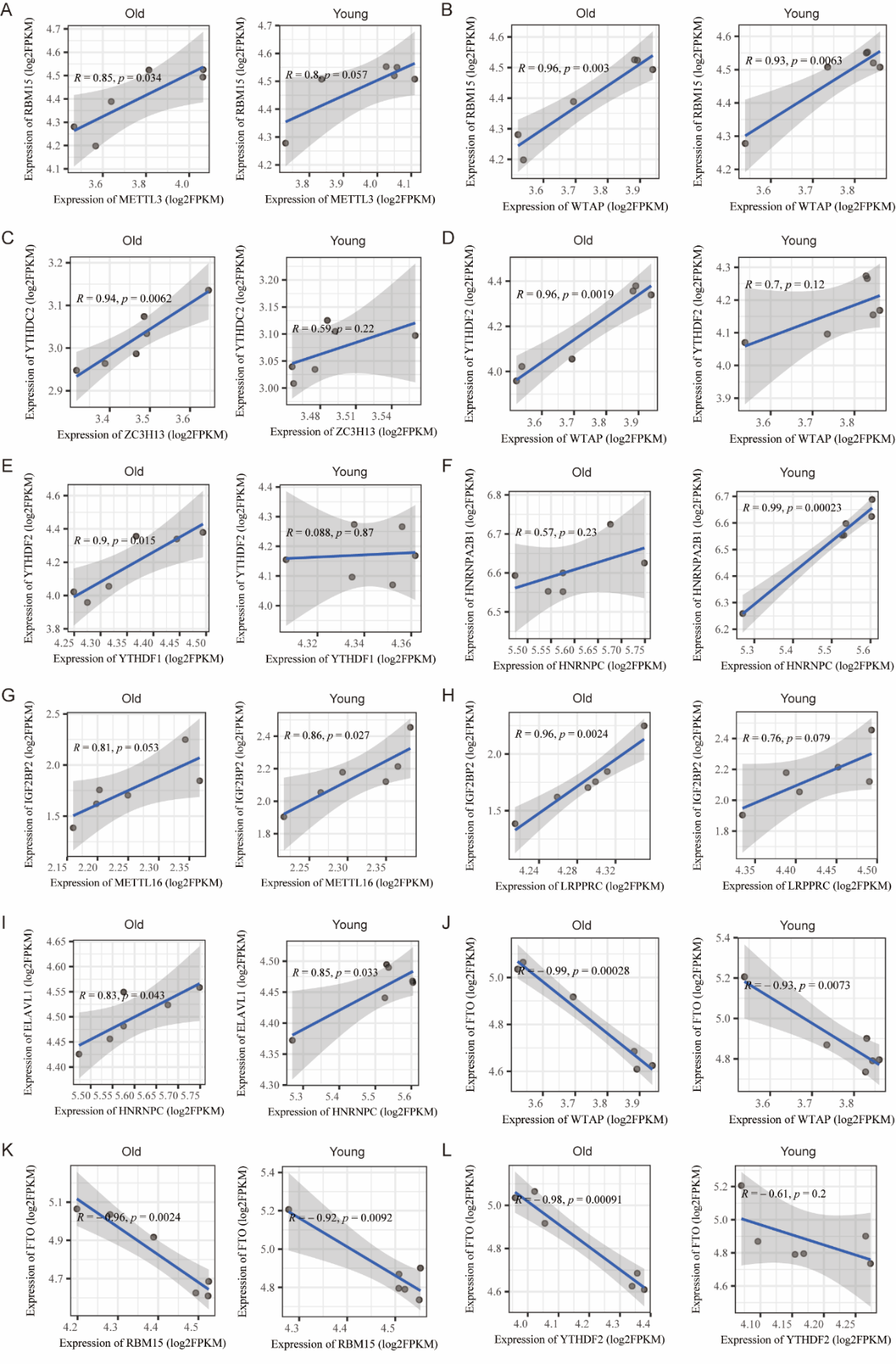


**Supplementary Figure 2** The expression of other m6A regulators in cochlea tissue by qPCR. n = 5; **p* < 0.05; ***p* < 0.01, ****p* < 0.001; ns, not significant; qPCR, Quantitative Polymerase Chain Reaction.

**
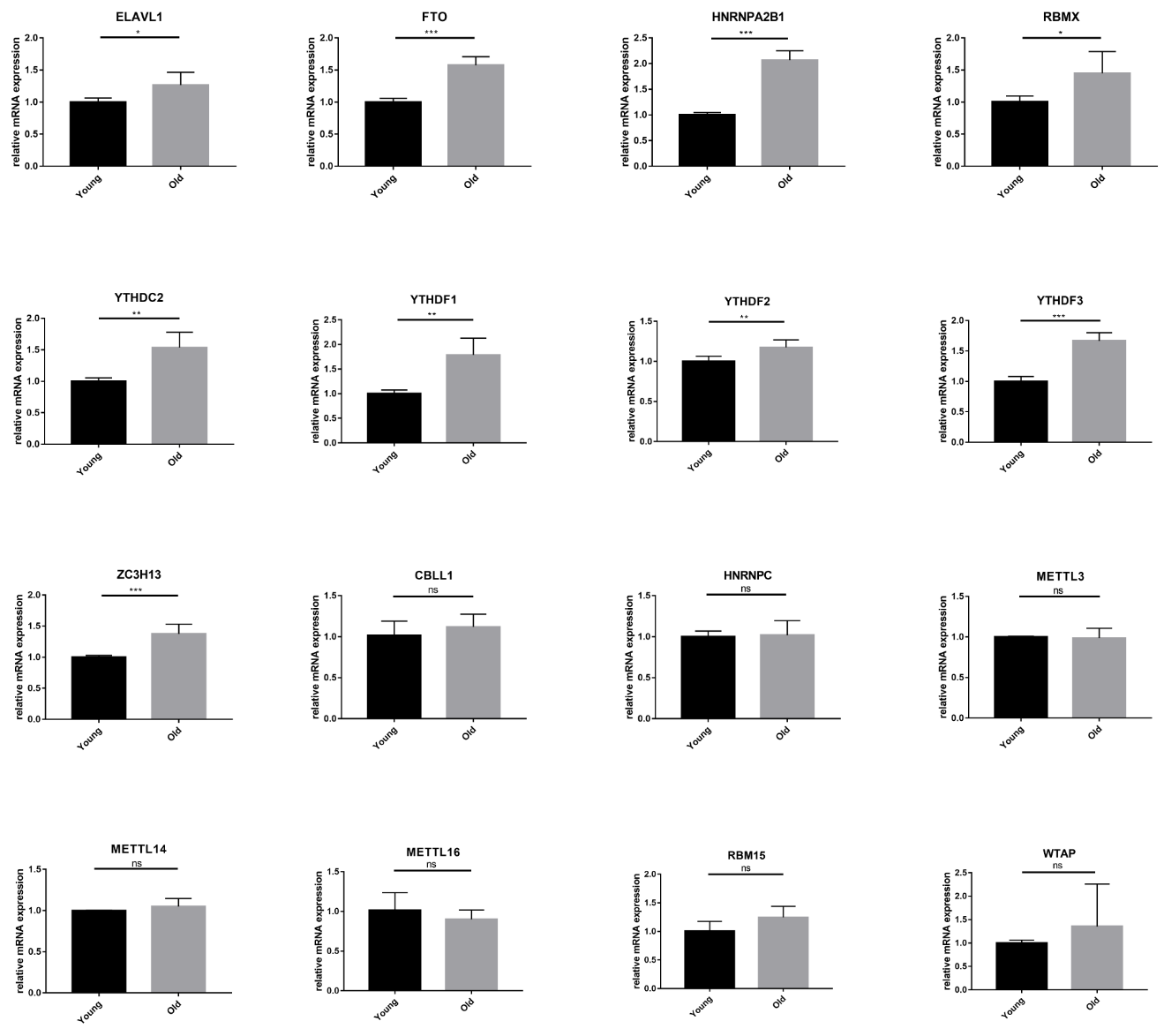
**
